# Perioperative periprosthetic femur fracture associated with direct anterior total hip arthroplasty using metaphyseal fit and fill stem

**DOI:** 10.1007/s00590-023-03682-z

**Published:** 2023-09-26

**Authors:** Frederic Washburn, Jacob Mushaben, Clayton Eichenseer, Brent Sanderson, Britni Tran, Thomas Golden

**Affiliations:** 1 Department of Orthopedics, Community Memorial Hospital, 147 Brent St., Ventura, CA 93003 USA; 2 Graduate Medical Education, Community Memorial Hospital, 147 Brent St., Ventura, CA 93003 USA

**Keywords:** Perioperative periprosthetic femur fracture, Direct anterior, Total hip arthroplasty, Metaphyseal fit, Fill stem

## Abstract

**Introduction:**

This study aims to identify radiographic and clinical risk factors of perioperative periprosthetic femur fracture associated with the direct anterior approach (DAA) using a metaphyseal fit and fill stem. We hypothesize stem malalignment with this femoral implant places increased stress on the medial calcar region, which leads to an increased risk of periprosthetic fracture.

**Methods:**

We compared patients with periprosthetic femur fractures following DAA total hip arthroplasty (THA) utilizing the Echo Bi-Metric Microplasty Stem (Zimmer Biomet, Warsaw, IN) to a cohort of patients who did not sustain a periprosthetic fracture from five orthopedic surgeons over four years. Postoperative radiographs were evaluated for stem alignment, neck cut level, Dorr classification, and the presence of radiographic pannus. Univariate and logistic regression analyses were performed. Demographic and categorical variables were also analyzed.

**Results:**

Fourteen hips sustained femur fractures, including nine Vancouver B2 and five AG fractures. Valgus stem malalignment, proud stems, extended offset, and patients with enlarged radiographic pannus reached statistical significance for increased fracture risk. Low femoral neck cut showed a trend toward statistical significance.

**Conclusion:**

Patients undergoing DAA THA using a metaphyseal fit and fill stem may be at increased risk of perioperative periprosthetic fracture when the femoral stem sits proudly in valgus malalignment with extended offset and when an enlarged pannus is seen radiographically. This study identifies a specific pattern in the Vancouver B2 fracture cohort with regard to injury mechanism, time of injury, and fracture pattern, which may be attributed to coronal malalignment of the implant.

## Introduction

After first being described by Smith and Peterson in 1917 and through its mainstream orthopedic popularization in the 1980s and 1990s, the direct anterior approach (DAA) to the hip has become one of the more popular surgical approaches utilized during primary total hip arthroplasty. The trend in the last 10–15 years toward outpatient surgery for joint reconstruction and same-day hospitalization has only advanced the popularity of this approach, with over 55% of surgeons now saying they employ the DAA during total hip arthroplasty [1]. Several research studies have been published demonstrating higher patient satisfaction, lower levels of postoperative pain on the first day, and higher functional scores at 6 weeks and 3 months postoperatively [2, 3]. However, other publications have stated these differences are marginal and long-term data show no significant differences in outcome, functionality, or patient satisfaction. There is also a steep learning curve to the DAA. Most authors and publications suggest that about 100 DAA hip arthroplasty cases are needed to be proficient in the approach. Overall when comparing the complication rate of DAA versus the traditional posterior approach, it is similar in most aspects with the DAA having a 1.9% complication rate over the first 90 days. [4]

Of these complications, several studies have indicated the risk of intraoperative or postoperative femur fracture at about 1–3% [4–6]. Most of these occur in the calcar region and occur during broaching, trial stem placement, and most commonly, during final implant placement. Known risk factors for intraoperative femur fracture are female sex, age > 65, and the use of a cementless stem [7]. It has also been demonstrated that other approaches to the hip, such as the Hardinge [direct lateral] approach or the anterolateral approach, are independent risk factors for intraoperative femur fracture compared to the posterior approach [8, 9]. Recently, more literature has begun to look at the newer fit and fill stems and their association with complications surrounding THA. One study found that greater canal fill and coronal plane malalignment with DAA were significant risk factors for Vancouver B-type fractures of the femur, with 98% of those fractures occurring postoperatively at an average of 44 days. [10] However, other studies have conflicting data, showing that fit and fill stems may be associated with a lower risk of perioperative fracture, although stems without a collar were associated with a higher risk [11]. Most of the time, femoral shaft fractures just distal to or around the implant are often well managed with good outcomes through a definitive treatment algorithm that includes cabling, revision arthroplasty, or open reduction internal fixation. [12–14]

This study aims to identify radiographic and clinical risk factors of perioperative periprosthetic femur fracture associated with the direct anterior approach using a metaphyseal fit and fill stem. We hypothesize stem malalignment with this femoral implant places increased stress on the medial calcar, which leads to an increased risk of periprosthetic fracture.

## Methods

This is a retrospective internal comparison cohort study of patients with DAA THA using the Echo Bi-Metric Microplasty Stem (Zimmer Biomet, Warsaw, IN). Five orthopedic surgeons performed the procedure over 4 years, with the total number of patients being 286 undergoing the DAA in that span. All orthopedic surgeons are either joint arthroplasty fellowship-trained or general orthopedic surgeons who perform a high volume of joint arthroplasty, utilizing DAA as their primary total hip surgical approach. Patients with a primary diagnosis of periprosthetic femur fractures following DAA THA using the Echo Bi-Metric Microplasty Stem (Zimmer Biomet, Warsaw, IN) (*N* = 14) were compared to patients (*N* = 272) who underwent the same approach with the same implant and did not sustain a fracture. DAA is predominantly used at our institution and does not have any absolute contraindications. However, patients with severe morbid obesity (BMI > 40 kg/m^2^) or significant pannus with skin changes (i.e., *candida* infection) in their inguinal fold are more likely to undergo a posterior approach. Bone quality and femoral geometry did not factor into surgical decision to use a DAA. Patient data were reviewed in the hospital's electronic medical records as needed, including paper charts, surgical schedules, and reports. Patient demographics and hospital data, including age, sex, weight, body mass index (BMI), sex, side, implant type, implant size (manufacturers’ listed size), history of previous hip surgery, preoperative ambulatory status (full, cane, walker, wheelchair), length of hospital stay, as well as disposition to home or skilled nursing facility were recorded. Patients were identified as having undergone THA by CPT coding and preoperative diagnosis codes, and operative reports were reviewed to identify those who had undergone arthroplasty through the DAA to the hip. All patients identified in this manner were also noted to have osteoarthritis of the affected hip.

The initial postoperative anteroposterior pelvis radiographs with the lower limbs internally rotated approximately 15° were evaluated for femoral stem alignment, femoral neck cut level, stem position in relation to the neck cut, Dorr Classification, and measure of obesity quantified by the radiographic presence of a pannus on the AP pelvis x-ray. Radiographic analysis was independently performed by two investigators utilizing a standardized protocol. Stem position and alignment were agreed upon in approximately 85% of cases, and any discrepancies in the analysis were resolved via a third independent investigator. Femoral stem alignment was characterized as neutral, varus, or valgus, measured by drawing a line parallel to the femoral canal axis and determining the position of the stem in relation to that line. The angle between the femoral canal and the stem was then measured, with a qualification of valgus or varus being three degrees or greater. The femoral neck cut level was determined by measuring the amount of femoral neck remaining above the lesser trochanter. Preoperative assessment and templating to determine neck offset in cases in the study showed an average templated neck length of 1 cm remaining above the lesser trochanter. A neck cut considered low was < 5 mm of femoral neck remaining above the lesser trochanter. The normal neck cut was between 5 and 15 mm of femoral neck remaining. A high neck cut was > 15 mm of femoral neck remaining. Stem position in relation to the neck cut was characterized as flush, proud (stem positioned above the femoral neck), or subsided (stem positioned below the femoral neck). The Dorr Classification was graded as either A, B, or C. The radiographic presence of pannus was measured as 1, 2, or 3 based on whether pannus opacity was seen above the pubic symphysis, between the upper and lower borders, or below the pubic symphysis. Additionally, implant variables were evaluated, including femoral stem size, femoral head size, acetabular cup size, and neck offset.

To identify radiographic and clinical risk factors of perioperative periprosthetic femur fracture associated DAA using a metaphyseal fit and fill stem, bivariate logistic regression with dependent variable fracture was run to establish individual correlation, and results were used to aid in model selection. A multiple logistic regression model was then constructed with the dependent variable fracture to calculate adjusted odds ratios. Variables selected for this model had p-values < 0.1 in the bivariate analysis. Data analyses were done using the R-open source package. An Internal Review Board approved this study. There was no funding involved.

## Results

There were a total of 286 subjects with complete data for all variables, with 14 who experienced a hip fracture (Vancouver B2 = 9; greater trochanteric (GT) = 5) after the procedure. Four of these fractures were non-displaced and were managed non-operatively. One intraoperative greater trochanteric fracture was treated with open reduction internal fixation during the primary THA procedure secondary to fragment size and fracture displacement. All Vancouver B2 fractures occurred between 14 and 50 days status-post hip arthroplasty surgery. Three of these fractures occurred after a fall, and six occurred without a history of falls or trauma. All Vancouver B2 fractures were treated with Arcos Modular Femoral Revision Stem (Zimmer Biomet, Warsaw, IN) and cables.

**Fig. 1:**
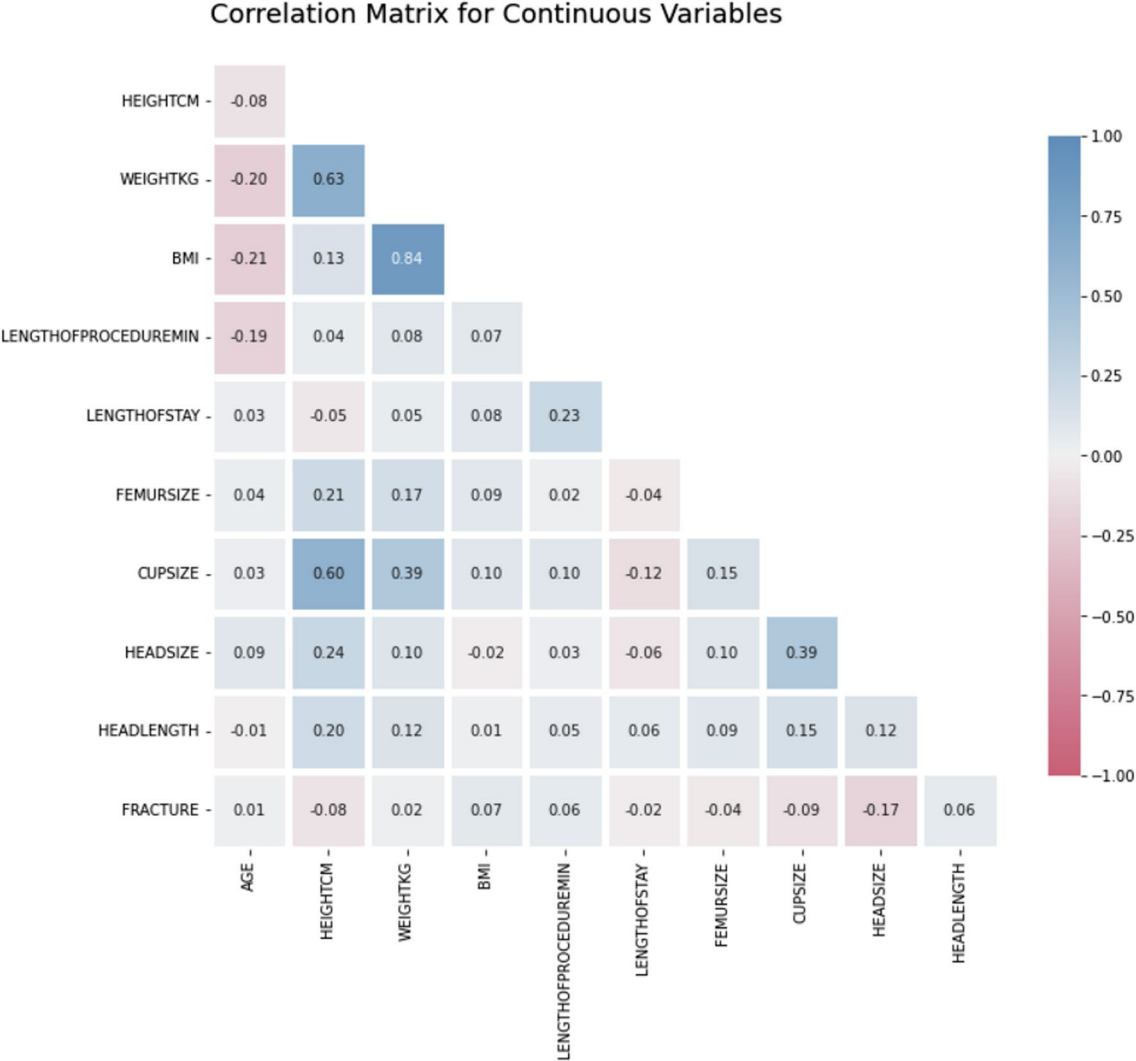
Correlation matrix

A correlation matrix was constructed to investigate the correlation between continuous variables and help inform variable selection for the analysis. BMI, Weight, and Height evidenced a high correlation, and for the remainder of the analysis, only Weight and Height were used. Additionally, Cup Size was correlated with Height, Weight, and Head Size. Fracture had the highest correlations with Height and Head Size among the continuous variables (Fig. 1).

Univariate logistic regressions and Fisher's Exact Tests (for categorical variables) between fracture and each variable were then run to determine the extent of correlation between fracture and each variable and statistical significance. This analysis resulted in Offset (Standard = 1), Head Size, Stem (Valgus = 1), Cut Location (Flush = 1), and Pannus (Skinny = 1) being statistically significant at 0.05 level. All significant variables had a protective effect on fracture except for the stem. Neck (Normal = 1) and Femur Size had p-values below 0.1 but above 0.05. These variables also had a protective effect on Fracture Status. Extended offset, smaller head size, valgus alignment of stem, proud stem, and fat pannus sign statistically significant (*P* < 0.05) for increased fracture risk. Low neck cut and smaller femur size demonstrated a trend toward statistical significance to increased fracture but failed to reach statistical significance (Table 1).

**Table 1:** Univariate logistic regressions (Fisher’s exact test to confirm categorical variables)

Variable	OR	*P*-value	Lower CI (5%)	Upper CI (95%)
Age	1.01	0.80	0.95	1.07
Height	0.96	0.19	0.90	1.02
Weight	1.00	0.77	0.98	1.03
BMI	1.07	0.22	0.96	1.18
Discharge (SNF = 1)	2.29	0.22	0.60	8.67
Smoking status (yes = 1)	0.00	1.00	0.00	Inf
Operative side (right = 1)	1.65	0.38	0.54	5.04
Length of procedure	1.01	0.35	0.99	1.02
Length of stay	0.88	0.71	0.45	1.73
Offset (standard = 1)**	**0.05**	**0.01**	**0.01**	**0.41**
Femur size*	**0.78**	**0.10**	**0.59**	**1.05**
Cup size	0.89	0.13	0.77	1.03
Head size**	**0.61**	**0.01**	**0.43**	**0.87**
Head length	1.08	0.35	0.92	1.27
ASA class (very sick)	2.09	0.18	0.70	6.19
STEM (valgus = 1)**	**3.25**	**0.04**	**1.06**	**9.96**
Cut location (flush = 1)**	**0.14**	**0.01**	**0.03**	**0.63**
Neck (normal = 1)*	**0.32**	**0.06**	**0.10**	**1.03**
DORR (*B* = 1)	0.64	0.44	0.21	1.96
DORR (*C* = 1)	2.02	0.53	0.22	18.48
Pannus (skinny = 1)**	**0.24**	**0.01**	**0.08**	**0.72**

Bold values are statistically significant

*P-value below 0.1

**Significant at 0.05 alpha level

Two logistic regression analyses were run to determine significant effects on Fracture Status. The first logistic regression only included variables with a p-value less than 0.1, and the second had all variables. The first analysis resulted in Offset (Standard = 1), Head Size, and Pannus (Skinny = 1) being significant at 0.05. Stem (Valgus = 1) had a p-value of 0.058 but failed to achieve statistical significance at a 0.05 level. The odds of fracture for patients with Standard Offset are 98% lower than those with Extended Offset. The odds of fracture for patients with a one-unit increase in Head Size are 80% lower. The odds of fracture for a skinny stem patient are 49% lower than for a Stem non-skinny (fat and fatter) patient (Table 2). The analysis with all variables included had Offset (Standard = 1), Pannus (Skinny = 1), and Head Length significant at 0.05. Cut Location (Flush = 1) and Operative Side (Right = 1) had p-values below 0.1 but failed to be statistically significant at a 0.05 level (Table 3).

**Table 2:** Multiple logistic regression: significant univariate variables

Variable	OR	*P*-value	Lower CI (5%)	Upper CI (95%)
Offset (standard = 1)**	**0.02**	**0.00**	**0.00**	**0.31**
Cut location (flush = 1)	0.27	0.12	0.05	1.41
Neck (normal = 1)	0.47	0.27	0.12	1.82
STEM (other = 1)*	**3.69**	**0.06**	**0.96**	**14.26**
Pannus (skinny = 1)**	**0.20**	**0.02**	**0.05**	**0.81**
Head size**	**0.51**	**0.03**	**0.29**	**0.92**

Bold values are statistically significant

*P-value below 0.1

**Significant at 0.05 alpha level

**Table 3:** Multiple logistic regression: all variables

Variable	OR	*P*-value	Lower CI (5%)	Upper CI (95%)
Discharge (SNF = 1)	1.86	0.57	0.21	16.35
Operative side (Right = 1)*	**4.69**	**0.09**	**0.77**	**28.44**
Offset (standard = 1)**	**0.00**	**0.01**	**0.00**	**0.22**
Cut location (flush = 1)*	**0.16**	**0.06**	**0.02**	**1.08**
ASA class (very sick = 1)	1.08	0.93	0.20	5.90
STEM (other = 1)	2.86	0.19	0.60	13.74
Neck (normal = 1)	0.41	0.28	0.08	2.09
DORR (B = 1)	0.56	0.53	0.09	3.41
DORR (C = 1)	0.14	0.57	0.00	112.63
Pannus (skinny = 1)**	**0.06**	**0.03**	**0.00**	**0.78**
Age	1.00	0.91	0.92	1.07
Height	1.02	0.72	0.90	1.17
Weight	0.99	0.69	0.92	1.06
Head length	0.68	0.21	0.37	1.24
Cup size	0.99	0.95	0.71	1.38
Head size	0.60	0.29	0.23	1.55
Head length**	**1.25**	**0.04**	**1.01**	**1.55**
Femur size	0.68	0.21	0.37	1.24

Bold values are statistically significant

*P-value below 0.1

**Significant at 0.05 alpha level

## Discussion

**Fig. 2:**
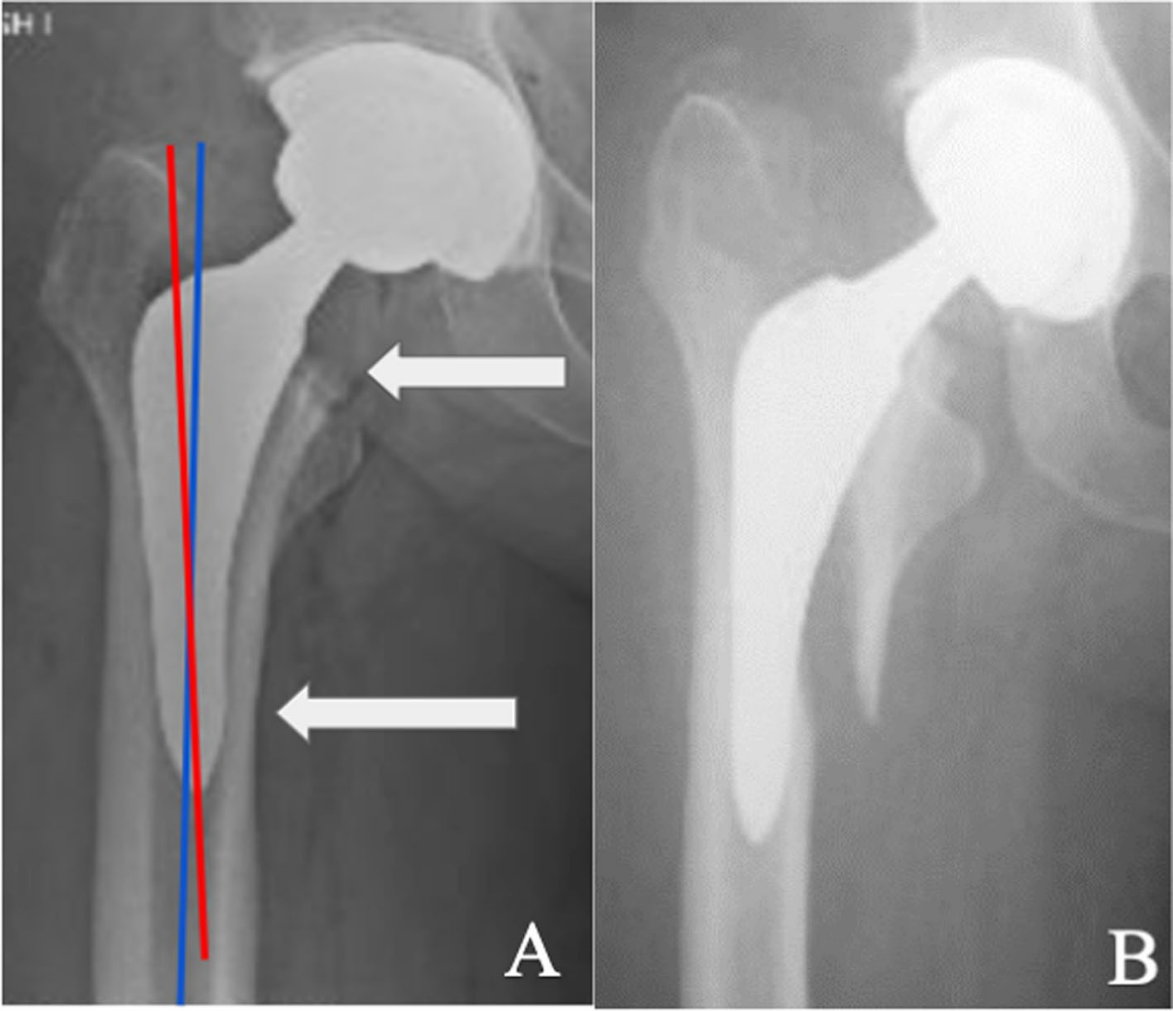
Figure **A** (left) demonstrates the initial postoperative radiograph with an extended offset femoral stem in slight valgus malalignment, a low femoral neck cut, and the stem positioned proud in relation to the femoral neck cut. Figure **B** (right) demonstrates the resulting Vancouver B2 fracture with the apex of the medial proximal femur fragment proximal to the stem tip

**Fig. 3:**
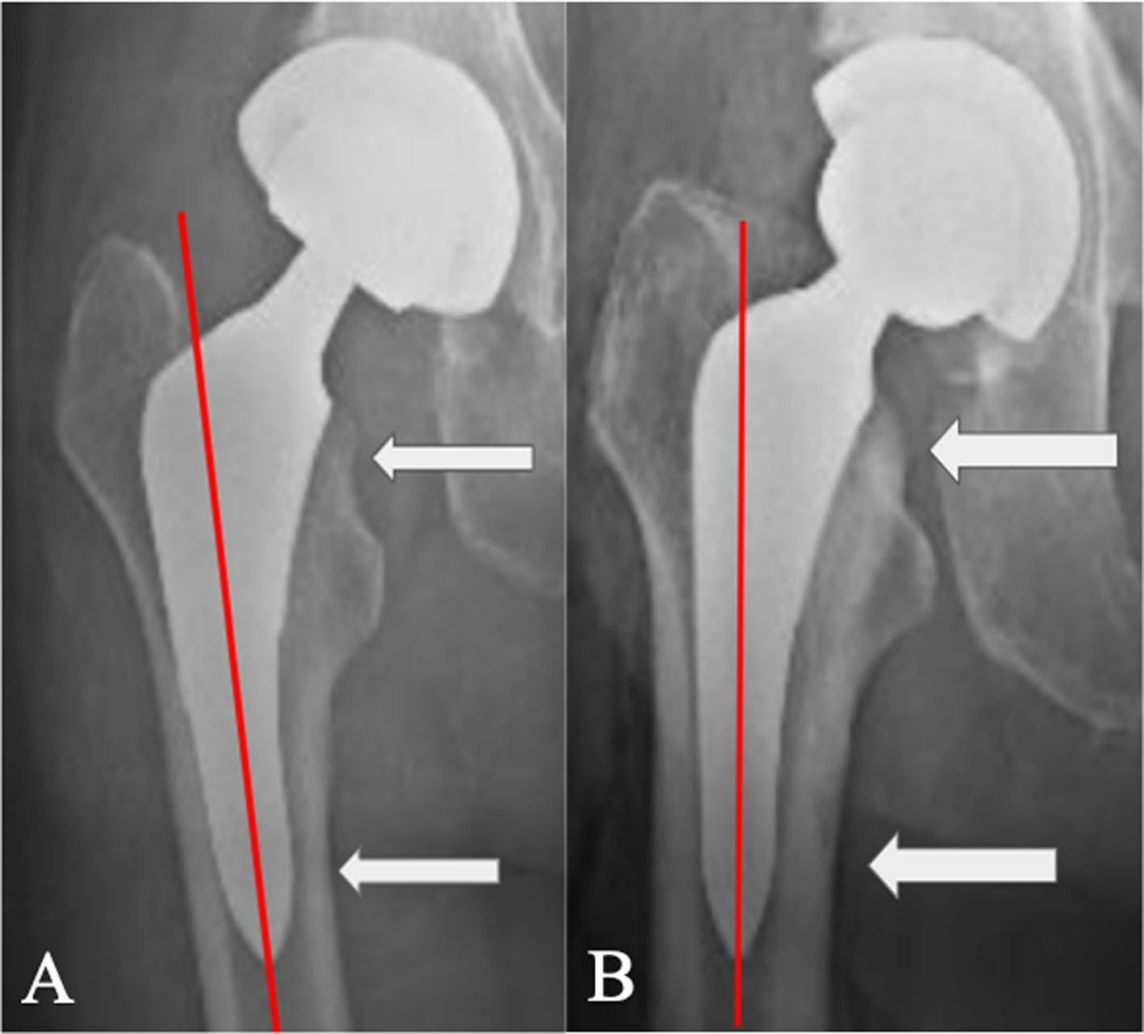
Image **A** (left) and **B** (right) demonstrate femoral stem prostheses positioned in line with the femoral intramedullary axis, with a normal neck cut level, and with the stem flush with the femoral neck cut

This study demonstrates patients undergoing THA utilizing a DAA with a metaphyseal fit and fill stem may be at increased risk of perioperative periprosthetic femur fracture when the femoral stem is in valgus malalignment, the femoral stem is inserted proud in relation to the femoral neck cut, an enlarged pannus is seen radiographically, and when an extended offset implant is used. We discovered a similar pattern in the Vancouver B2 fracture cohort, which may be attributed to implant malalignment (Fig. 2). The Vancouver B2 cohort suffered fractures between 2 and 6 weeks postoperatively. Patients gradually increase their activity level during this period, but bony ingrowth into the porous coated stem is still occurring. One author noted nearly all institutional Vancouver B2 fractures occurring at their institution occurred within 33 days. [15] Six of the nine (66%) Vancouver B2 fractures in this study occurred without a fall history. This could be a result of an intraoperative fracture that was too subtle to be appreciated on intraoperative fluoroscopy or not completed, leading it to be unnoticed when placing the final implant with subsequent failure. Prior research has shown that up to 40% of intraoperative fractures may be missed during the surgery; however, none of those missed fractures were seen in the calcar region [16]. All six patients described a twisting mechanism while ambulating, thus creating a combined rotational and axial force on the proximal femur. A 2020 study demonstrated femoral stems without a collar required a significantly smaller amount of torsional force to fracture the calcar and propagate distally compared to a femoral stem with a collar [17]. The force required was a difference of nearly 30Nm and was always a spiral pattern. The fracture pattern was also consistent among the Vancouver B2 patients, where a medial fracture fragment was generated with the fracture apex proximal to the stem tip. This finding may be explained by coronal malalignment of the metaphyseal fit and fill stem. The press-fit metaphyseal fit and fill stem is designed to achieve circumferential contact with the proximal femoral metaphysis. Proper stem positioning requires broaching and stem implantation down the axis of the femoral canal (Fig. 3). Our hypothesis is that intraoperative broaching and stem implantation in valgus pots the stem into the medial femoral cortex, which causes the stem to sit proud in relation to the femoral neck cut. This creates a stress riser or an occult fracture in the region where the fracture apex occurs. Repetitive axial loading and rotational forces during the perioperative period prior to completing bony ingrowth into the prosthesis may weaken this area of bone and result in fracture at the medial femoral cortex just proximal to the stem tip. A mismatch in sagittal alignment of the stem could have been another potential risk factor for proximal femoral fracture, much the same as the coronal malalignment. Sagittal malalignment has been previously associated with higher risk of aseptic loosening in tapered stems; however, this radiographic parameter was not assessed in this study. [18]

The choice of femoral offset was also a statistically significant risk factor for perioperative femoral fracture in this study, as 13 of 14 (93%) patients in the fracture cohort were recipients of high offset neck implants. This risk factor has not been previously reported for cementless or tapered stems. However, a large retrospective meta-analysis by Lamb et al. in 2020 demonstrated high offset in long cemented stems was correlated with an increased fracture risk after primary THA [19]. Prior biomechanical studies have shown that the restoration of femoral offset is critical to restoring function of the abductor lever arm for gait mechanics after THA [20]. However, this also leads to increased joint reactive forces, which when transferred across the bone-implant interface, may lead to an increased risk for fracture [21]. This study demonstrates the expected odds ratio of fracture for patients with standard offset necks is 98% lower than for patients with extended offset. Obesity is also a well-known risk factor in arthroplasty surgery, and this study further demonstrated that the presence of a pannus sign is an independent risk factor for perioperative femur fracture when utilizing the DAA for THA [22–24]. The odds of fracture for an obese patient are 51% higher than that of a skinny patient (i.e., one without a Pannus Sign present on the AP radiograph). While current literature also demonstrates female gender is a risk factor for perioperative fracture, we did not appreciate this in our study.

**Fig. 4:**
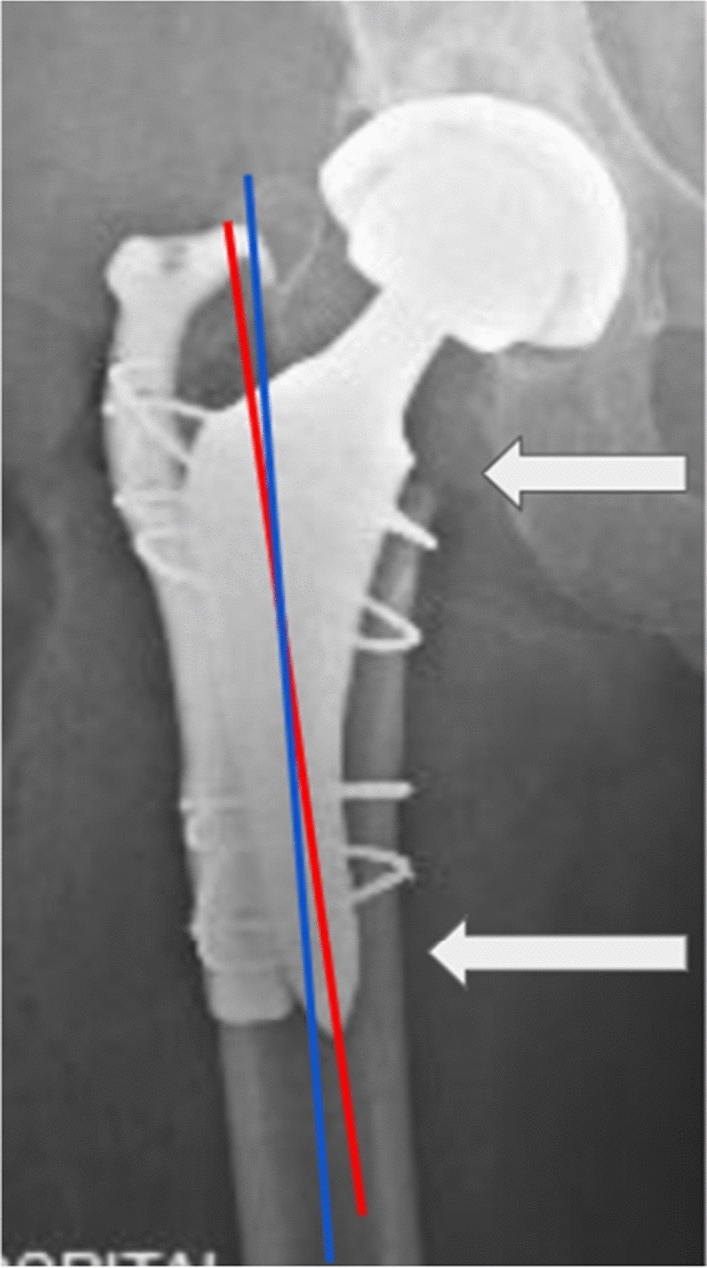
AP radiograph of the right hip resulted in an intraoperative displaced greater trochanter fracture managed with a proximal femur plate and cables. The stem is in significant valgus in relation to the femoral intramedullary axis

All five of the greater trochanteric fractures included in this study were retrospectively found to have occurred intraoperatively. Intraoperative fluoroscopy was utilized in every case to confirm implant position, but it was unable to detect very subtle non-displaced fractures in four of five of the greater trochanteric fracture cases at the time of the procedure. The four of five subtle non-displaced fractures were found on review of postoperative radiographs with clinical correlation of pain over the greater trochanter. These were treated conservatively with weight bearing as tolerated and limited active abduction. For the one remaining greater trochanteric fracture, intraoperative fluoroscopy revealed significant displacement, which required internal fixation during the index THA. Coronal malalignment of the femoral component may explain these fractures as well. The direct anterior approach positions the femoral canal vertically during femoral implant preparation. Dropping one's hand during broaching creates a valgus trajectory, and forceful removal of the broach may cause the proximal-lateral aspect of the broach to contact the base of the greater trochanter, resulting in fracture. Furthermore, the stem-neck junction is shifted laterally with a valgus broaching trajectory, resulting in closer proximity to the greater trochanter. This increases the chance of contact between the calcar reamer and the greater trochanter, possibly resulting in fracture. In our patient with a displaced greater trochanter fracture that required internal fixation, the stem was placed in the valgus and the fracture occurred during the calcar reaming process (Fig. 4). The other four non-displaced greater trochanteric fractures occurred during the removal of the broach. All greater trochanteric femur fractures went on to heal uneventfully.

We recognize there are several limitations to this study. First, this study was a retrospective review of cases at a single institution in a community setting. Therefore, these results may not be generalizable to a larger population. However, this allows for reproducibility regarding operating parameters, procedure and operative technique, and hospital environment. The number of patients included in the study is relatively small, with an N of 286 and 14 sustaining some type of perioperative fracture; this number does not have a large power and may allow for some misrepresentation of data.

## Conclusion

This study identifies important risk factors associated with perioperative periprosthetic femur fractures for THA cases that utilize the DAA and a metaphyseal fit and fill stem. These include coronal valgus malalignment of more than three degrees, as well as extended offset stems and increased BMI. Future studies with higher power could further investigate this pattern to tailor the femoral preparation operative technique to decrease the fracture risk.

## Data Availability

Available upon request.
